# Introduction of Hypermatrix and Operator Notation into a Discrete Mathematics Simulation Model of Malignant Tumour Response to Therapeutic Schemes *In Vivo.* Some Operator Properties

**DOI:** 10.4137/cin.s2712

**Published:** 2009-10-21

**Authors:** Georgios S. Stamatakos, Dimitra D. Dionysiou

**Affiliations:** *In Silico* Oncology group, Laboratory of Microwaves and Fibre Optics, Institute of Communication and Computer systems, school of electrical and Computer engineering, national Technical University of Athens, GR-157 80 Zografos, greece

**Keywords:** cancer multiscale modeling, computer models, in silico oncology, tumour growth, tumour response to treatment, oncosimulator, radiobiology, operator notation, hypermatrix

## Abstract

The tremendous rate of accumulation of experimental and clinical knowledge pertaining to cancer dictates the development of a theoretical framework for the meaningful integration of such knowledge at all levels of biocomplexity. In this context our research group has developed and partly validated a number of spatiotemporal simulation models of *in vivo* tumour growth and in particular tumour response to several therapeutic schemes. Most of the modeling modules have been based on discrete mathematics and therefore have been formulated in terms of rather complex algorithms (e.g. in pseudocode and actual computer code). However, such lengthy algorithmic descriptions, although sufficient from the mathematical point of view, may render it difficult for an interested reader to readily identify the sequence of the very basic simulation operations that lie at the heart of the entire model. In order to both alleviate this problem and at the same time provide a bridge to symbolic mathematics, we propose the introduction of the notion of *hypermatrix* in conjunction with that of a *discrete operator* into the already developed models. Using a radiotherapy response simulation example we demonstrate how the entire model can be considered as the sequential application of a number of discrete operators to a hypermatrix corresponding to the dynamics of the anatomic area of interest. Subsequently, we investigate the operators’ commutativity and outline the “summarize and jump” strategy aiming at efficiently and realistically address multilevel biological problems such as cancer. In order to clarify the actual effect of the composite discrete operator we present further simulation results which are in agreement with the outcome of the clinical study RTOG 83–02, thus strengthening the reliability of the model developed.

## 1. Introduction

An inelastic prerequisite for an effective treatment of cancer is understanding and modeling the corresponding spatiotemporal natural phenomenon of tumour growth and response to therapeutic schemes concurrently on several *biocomplexity levels*. The usually fast growth and resilience of tumours suggest that they are emerging, opportunistic systems rather than random, disorganized and diffuse cell masses.^1^,^2^ Therefore, the entire *in vivo* growing tumour rather than only a single cell^3^ must be investigated and treated as a self‐organizing complex dynamic system. In this context there is need for advanced computational models to simulate the complexity of solid tumour growth, invasion and metastasis combining a range of disciplines including medical, biological, biophysical, engineering and statistical physics research.^4^

This section provides a brief outline of several of the concepts and earlier research efforts to model tumour behaviour. Duechting et al^5^ introduced a simulation model which concerns the *in vitro* case or the early avascular stages of small *in vivo* tumours and is based on a consideration of the distinct phases of the cell cycle. Kocher et al^6^,^7^ presented a simulation model of the development of a tumour spheroid and its response to radiosurgery. The detailed imaging based geometry of the clinical tumour which might facilitate the direct clinical validation of the model had not been considered however. Instead an equivalent spherical tumour was considered in place of the generally arbitrarily shaped actual tumour. Additionally, detailed cell cycle phase biology (phases G_1_, S, G_2_, M) had not been taken into account, grouping of the cells into only proliferating and dormant classes being considered instead. It is noted that none of the above mentioned models has been applied to large clinical tumours (of varied geometrical shapes) and none of them simulates shrinkage for an arbitrarily shaped clinical tumour undergoing treatment. In the pure tumour growth models presented by Kansal et al^1^,^2^ a discretising grid is used in which each geometrical cell is able to contain a large number of biological cells, although the grid has not been used to discretise clinical tumours of arbitrary shape. Swanson et al,^8^,^9^ Mandonnet et al,^10^ have developed clinically oriented spatiotemporally models of tumour growth and invasion concerning glioblastoma multiforme (GBM). Although growth and invasion constitute fundamental phenomena related to GBM treatment optimization, the investigators have not focused neither on the radiobiological nor on the pharmacodynamic mechanisms that determine the cell survival probabilities and the subsequent shrinkage. Byrne et al^11^ and Alarcon et al,^12^–^14^ have developed mathematical models of avascular tumour growth and angiogenesis evolution pertinent mainly to the initial stages of tumour development. Valuable insight can be gained using such models, but extension to clinical voluminous tumours is not an a priori manageable task. Wise et al^15^ have developed a three dimensional multi‐species non linear tumour growth model. A review of significant efforts to model cancer *in silico* (=*on the computer*) has also been presented by Deisboeck et al.^16^

An effort to overcome some of the above mentioned limitations has been previously made by our research group through the development of four‐dimensional patient-specific *in vivo* simulation models of *imageable* tumour response to radiotherapeutic and chemotherapeutic schemes.^17^–^29^ All parameters used in the models have already been defined and can be determined (in principle) experimentally or clinically. Therefore, use of new mathematically dictated parameters of ambiguous physical meaning has been avoided.

Looking now at the problem of cancer from a broader theoretical perspective we realize that the impressive rate of production of experimental and clinical knowledge pertaining to the disease dictates the development of a generic and desirably universal theoretical framework for the meaningful integration of such knowledge. The obvious reasons for knowledge integration are both deeper understanding of cancer and optimization of therapeutic schemes on the patient individualized context by performing *in silico* experiments. At the same time as cancer is an excellent paradigm of multilevel biological phenomena any rigorous theoretical treatment of cancer dynamics could provide important hints for the modeling and simulation of other biological phenomena including other homeostatic imbalances (diseases).^22^ As more and more complexity aspects of the *natural phenomenon* of cancer are incorporated into mathematical and computational models important discrete mathematics modeling treatments tend to become difficult to understand by the wider cancer modeling community. Therefore, the need for a symbolic mathematical notation has become obvious. Such an approach could be viewed as following the formalist school founded by D. Hilbert.^30^ According to the formalist thesis mathematics is concerned with *formal symbolic systems*.

Stimulated by these remarks we present the introduction of an operator notation into the malignant tumour response to therapeutic schemes *in vivo* making use of several versions of the simulation model developed by our group (see citations above). In order to proceed to the introduction of a discrete mathematics operator notation the anatomic region of interest and its biological dynamics are represented by a hypermatrix *ᾱ*. A *hypermatrix* can be viewed as a matrix of [a matrix of [… of [matrices (or vectors) ]…]]. The hypermatrix *ᾱ* is created by the superposition of a discretization mesh on the anatomic region of interest and the consideration of equivalence classes within each geometrical cell of the mesh representing the various phases within or out of the cell cycle that a biological cell can be found. Discrete time represents a further dimension of the hypermatrix.

## 2. Discretization of the Biological Problem

Collection of the appropriate imaging data (e.g. MRI T1 contrast enhanced, CT/PET, etc.), registration, interpolation and three dimensional reconstruction constitute the initial steps of an *in vivo* discrete mathematics treatment of the tumour growth and therapy response phenomenon. A discretizing mesh is superimposed on the anatomic region of interest and the contents of each geometrical cell of the mesh are distributed into equivalence classes corresponding to the various phases within or out of cell cycle. The mean time spent within each phase is another parameter that characterizes the subsets of each geometrical cell.^18^,^23^,^24^

## 3. Operator and Hypermatrix Notation

The following mathematical entities are considered in the proposed treatment: *ā* stands for the hyper‐matrix corresponding to and dynamically describing the anatomical region of interest (including the tumour and possibly the surrounding normal tissue). Each vector element of the hypermatrix *ᾱ* is considered to have the following form that corresponds to a discretization mesh geometrical cell:

(1) $$\underline{\bar{a}}(x_{i},y_{j},z_{k},p_{l},t_{n})=\left(g^{ijkln},N_{p}^{ijkln},t_{p}^{ijkln},h_{p}^{ijkln},{\tilde{h}}_{p}^{ijkln}\right)$$

(2) $$\begin{array}{l} \underline{\bar{a}}(t_{0})={\underline{\bar{a}}}_{0}\;\text{initial}\;\text{state of the tumour }(\text{just before }\text{the}\\ \text{start of the treatment course to be simulated}) \end{array}$$

where the following symbols have been introduced:

*p*: phase within or out of the cell cycle

*g*: oxygen and nutrient provision

*N_p_*: number of biological cells in phase *p*

*t_p_*: mean time spent in phase *p* (time is usually measured in *h*)

*h_p_*: number of therapy hit cells residing in phase *p*

*h̃*_p_ number of non therapy hit cells residing in phase *p*

(3) $$x_{i}\in [x_{\text{min}},x_{\text{max}}]$$

(4) $$y_{j}\in [y_{\text{min}},y_{\text{max}}]$$

(5) $$z_{k}\in [z_{\text{min}},z_{\text{max}}]$$

(6) $$t_{n}\in [0,t_{\text{max}}]$$

(7) $$p_{l}\in [G_{1},S,G_{2},M,G_{0},A,N,D]$$

where

*ξ*_min_, *ξ*_max_ denotes the minimum and maximum value respectively of the generic variable *ξ* during the simulation

*G*_1_ denote the *G*_1_ cell cycle phase,

*S* denotes the DNA synthesis phase,

*G*_2_ denotes the *G*_2_ cell cycle phase,

*M* denotes mitosis,

*G*_0_ denotes the dormant *G*_0_ phase,

*A* denotes the apoptotic phase,

*N* denotes the necrotic phase,

*D* denotes the remnants of dead cells,

(8) $$g\in \{s,\tilde{s}\}$$

*s* stands for suffcient oxygen and nutrient provision (for tumour cell proliferation), *s̃* stands for insuffcient oxygen and nutrient provision (for tumour cell proliferation).

Obviously this binary character of the oxygen and nutrient provision is to be considered only a first simplifying approximation.

(9) $$N_{p}\in N_{0}$$

*N*_0_ is the set of non negative integers

(11) $$t_{p}\in [0,t_{p\text{max}}]$$

(12) $$h_{p}\in [0,N_{p}]$$

(13) $${\tilde{h}}_{p}\in [0,N_{P}]$$

It should be noted that in general all physicobiologically different components of each hypermatrix element can be considered dependent on all five dimensions of the proposed abstract space of tumour dynamics. For example oxygen and nutrient provision can change dramatically in space and time within the tumour. The treatment outcome is generally dependent on the phase in which a cell resides when irradiated or treated with chemotherapy. Cell cycle phases have generally different durations and therefore the mean time spent within each phase equivalence class of a given geometrical cell is dependent on the cell phase. Even the oxygen and nutrient provision (to the biological cells belonging to the same phase within a geometrical cell) may be microscopically related to the phase under consideration. A relatively large number of tumour cells within the G_0_ phase located around a given point may imply inadequate oxygen and nutrient provision, although in this particular case dormancy is normally the outcome rather than the cause of inadequate oxygen and nutrient provision

**Figure 1:**
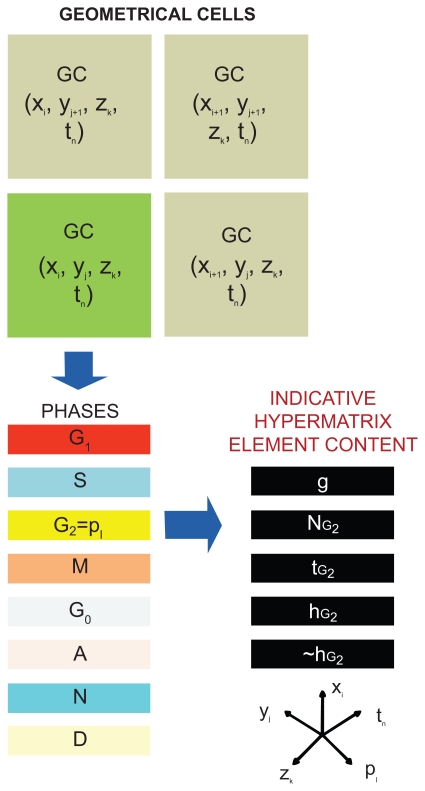
A schematic representation of equation 1 showing the location of an indicative hypermatrix element *ā* (*x_i_*, *y_j_*, *z_k_*, *p_l_*, *t_n_*) and its physically inhomogeneous and multidimensional content (*g^ijkln^*, *N_p_^ijkln^*, *t_p_^ijkln^*, *h_p_^ijkln^*, *h̃_p_^ijkln^*) [see text for symbols; ~*h_G2_* ≡ *h̃_G_*]. The proposed five dimensional discrete abstract space of tumour dynamics (corresponding to the localization of each hypermatrix element) is shown on the bottom right of the diagram. Three dimensions (corresponding to variables *x_i_*, *y_j_*, *z_k_*) represent space, another one (corresponding to variable *t_n_*) represents time and the fifth one (corresponding to variable *p_l_*) represents the cell phase within or out of the cell cycle in which a biological cell or a set of biological cells within a geometrical cell of the discretization mesh is found at a given instant.

Figure 1 provides a schematic representation of the proposed five dimensional discrete abstract space of tumour dynamics. Three dimensions (those corresponding to the variables *x_i_*, *y_j_*, *z_k_*) represent space, another one (corresponding to the variable *t_n_*) time and the fifth one (corresponding to the variable *p_l_*) represents the cell phase within or out of the cell cycle in which a biological cell or a set of cells within a geometrical cell of the discretization mesh is found at a given instant. The entire simulation can be viewed as the periodic application of a number of discrete algorithmic operators on the hypermatrix of the anatomic region of interest. The period of application is equal to the time separating two consecutive discretization mesh scans. This has been taken equal to 1h in all applications referred to in this particular paper.

The various modules of algorithmic manipulations on the hypermatrix can be thought of as corresponding to discrete operators acting on the hypermatrix in analogy to the action of continuous operators on a wave function in quantum mechanics (Schiff 31 pp. 148–186). The critical importance of considering abstract (vector) spaces and operators has been made clear by practically all fields of physics (Morse and Feshbach, 32 Part 1, pp. 76–92).

In order to proceed to a symbolic formulation of the operator application we make use of the following symbols:

*f* stands for the composite discrete operator i.e. the operator formed by the synthesis of all partial operators sequentially acting on the hypermatrix. Therefore, the updated hypermatrix at the time point *t_n_*_+1_ is given by

(14) $$f\left(\underline{\bar{a}}\left(t_{n}\right)\right)=\underline{\bar{a}}\left(t_{n+1}\right)$$

The composite operator can be written as

(15) $$f=f^{U}f^{E}f^{C}f^{H}f^{O}f^{T}$$

where

(16) $$f^{J},J\in \{U,E,C,H,O,T\}\;\text{stands for a}“\text{partial operator}”$$

*T* stands for time update (i.e. just the increase of time by e.g. 1 h and not the updated state of the hypermatrix *ᾱ* at any time *t_n_*).

*O* stands for the oxygen and nutrient provision status

*H* stands for the effect of therapy (mainly cell survival)

*C* stands for the eventually perturbed cell cycling due to therapy

*E* stands for differential expansion or shrinkage

*U* stands for oxygen and nutrients field update

(17) $$\begin{array}{l} f^{U}\left(f^{E}\left(f^{C}\left(f^{H}\left(f^{O}\left(f^{T}\underline{\bar{a}}\left(t_{n}\right)\right)\right)\right)\right)\right)\\ =\underline{\bar{a}}\left(t_{n+1}\right),n=0,1,\ldots .. \end{array}$$

or in a more compact writing:

(18) $$\begin{array}{l} f^{U}f^{E}f^{C}f^{H}f^{O}f^{T}\underline{\bar{a}}(t_{n})\\ =\underline{\bar{a}}\left(t_{n+1}\right),n=0,1,\ldots .. \end{array}$$

where the application of the operators takes place from the right to the left.

It is noted that the term *partial operator* as used in this work essentially denotes the application of complex algorithmic manipulations. The entire model has been constructed with a number of algorithmic manipulations (*partial operators*) applied repeatedly in a given order. Therefore, the whole model (entire algorithm) can be considered as the application of a composite operator. Subsequently this composite operator can always be decomposed into partial operators since the former is nothing more than a conceptual clustering of the latter.

It is quite obvious that the above mentioned concepts and symbols cannot include all the information needed for the simulation to run. Their role is (at least at the present stage) rather to identify and decompose the major conceptual mathematical treatment steps than to represent any assumption details. The proposed approach is to be seen as a continually evolving and optimized process. Examples of such evolutionary stages could be the following. In order to address the non imageable components of highly invasive tumours such as glioblastoma multiforme at a large time scale further operators could be proposed so as to explicitly handle diffusion phenomena at the cellular/tissue level. Additional operators could handle the refined biomechanics of the tumour and adjacent normal tissues as well complex molecular networks which largely determine the response of a single tumour cell to treatment (see Section 5).

## 4. Non Commutativity of the Operators

A careful study of the behaviour of any two of the “partial operators” *f^J^*, *f^K^* where *J*, *K* ∈ {*U*, *E*, *C*, *H*, *O*, *T* } reveals that they are *non commutative*. It is noted that the terms *commutative* and *permutable* are used interchangeably when applied to operators (but not always when applied to subgroups).^33^ For example applying the cell cycle clock ( *f^C^*) to the proliferating cells first and subsequently calculating the effect of a radiotherapy fraction (*f^H^*) may lead to a substantially larger number of surviving cells than the opposite sequence. The reason for such a difference could be the successful completion of mitosis for far more tumor cells in the first case which would lead to a larger number of tumour cells (tumour burden) before treatment is applied. Furthermore, it must be stressed that in general each partial operator has to be applied concurrently on all elements‐geometrical cells of the hypermatrix *ᾱ* (possibly already transformed by other partial operators) as there are in general interdependences among the various geometrical cells [e.g. differential shifting of the overflowing that have emerged following mitoses].

## 5. Multilevel Biology Considerations: The “Summarize and Jump” Strategy

In order to achieve a quite realistic prediction of the response of a tumour to therapeutic interventions several levels of biocomplexity have to be addressed at the same time. The decomposition of the composite discrete operator *f* to its constituent “partial operators” *f^J^*, *J* ∈{*U*, *E*, *C*, *H*, *O*, *T*} provides a conceptual tool useful for the analysis and superposition of several critical mechanisms that may take place on different biocomplexity levels. For example the molecular profile of a given tumour (e.g. the expressions of a number of critical genes) can be used in order to perturb the population based average survival fraction following irradiation with dose D so that the genetically produced radiosensitivity or radioresistance of the particular tumour of a given patient is taken into account during the simulation. This “summary” of the molecular level phenomena is currently incorporated into the partial operator *f^H^* which represents the microscopic effect of the treatment intervention on the tumour. One could interpret the procedure of perturbing the population based average values of the tumour biological parameters as a two step process. The first step refers to “summarizing” what is happening on one biocomplexity level (here the molecular level) and providing the amount of perturbation whereas the second one refers to the “jumping” to another level (here the cellular level). In this case the “summary” refers to the percentage by which the survival fraction will have to be perturbed whereas the “jumping” refers to the fact that the individualized survival fraction is related to the cellular level on which the main bulk of the simulation process takes place. Furthermore, it is worth noting that it is at the cellular level that complete success or failure of tumour treatment is defined since complete success of tumour treatment implies that not even a single proliferative or dormant tumour cell has been left alive.

## 6. Some Indicative Points of the Model and their Correspondence to Specific “Partial Operators”

The introduction of the suggested notation is demonstrated through a version of a simulation model of (*T1 gadolinium enhanced*) *imageable* glioblastoma response to radiotherapeutic schemes. As a thorough account of the assumptions made would be beyond the scope of the present paper we only provide an indication of the operator notation introduction by referring to selected modeling points. Further modeling details can be found i.a. in.^18^,^19^,^23^,^24^,^26^,^27^

In order to clinically validate the simulation model, a series of simulation executions corresponding to the various arms of the RTOG study 83‐02 have been performed.^34^ This was a randomized Phase I/II study of escalating doses for Hyperfractionated radiotherapy (HF, 1.2 Gy twice daily to doses of 64.8, 72, 76.8, or 81.6 Gy) and Accelerated Hyperfractionated radio‐therapy (AHF, 1.6 Gy twice daily to doses of 48 or 54.4 Gy) with carmustine (BCNU) for adults with supratentorial glioblastoma multiforme (GBM) or anaplastic astrocytoma. The study has revealed that GBM patients who received the higher HF doses had survival superior to the patients in the AHF arms or lower HF doses.

The *in silico* experiments performed involve three hypothetical *imageable* GBM tumours, otherwise identical except for their radiosensitivity parameters. In particular, the cases considered were the following:

- A GBM tumour with intact wild‐type (wt) p53 function, and accordingly adjusted LQ Model parameters.^35^
  $$\alpha_{p}=0.61\;{\text{Gy}}^{-1},\beta_{p}=0.02\;{\text{Gy}}^{-2}$$
- A GBM tumour with mutant (mt) p53 gene^35^:
  $$\alpha_{p}=0.17\;{\text{Gy}}^{-1},\beta_{p}=0.02\;{\text{Gy}}^{-2}$$
- A GBM tumour with intermediately adjusted radiosensitivity:
  $$\alpha_{p}=0.36\;{\text{Gy}}^{-1},\beta_{p}=0.02\;{\text{Gy}}^{-2}$$

In all cases, we set (Kocher;^7^ Perez and Brady,^45^ p. 99):

(19) $$\alpha_{G0}=\alpha_{p}/\text{OER},\beta_{G0}=\beta_{p}/{\text{OER}}^{2},\text{OER}=3$$

and

(20) $$\alpha_{S}=0.6\alpha_{p}+0.4\alpha_{G0},\beta_{S}=0.6\beta_{p}+0.4\beta_{G0}.$$

The meaning of the symbols used is the following:

*α_P_*, *β_p_*: the LQ Model parameters for all proliferative cell cycle phases except for the DNA synthesis phase (S phase).

*α_S_*, *β_S_*: the LQ Model parameters for the S phase.

*α_G_*_0_, *β_G_*_0_: the LQ Model parameters for the resting *G*_0_ phase.

The delivery of irradiation takes place at 08:00 and 16:00 every day, 5 days per week (no irradiation during weekends). The distribution of the absorbed dose in the tumour region is assumed to be uniform. It should also be noted that carmustin, which was administered to all patients enrolled in the RTOG—83-02 study, is assumed not to significantly modify the relative effectiveness of the radiation therapy schedules considered, as the chemotherapy administration schedule was the same for all patients.

### 6.1. Construction of the hypermatrix

The first process that takes place before the application of the operators is the discretization of the anatomic region of interest and the construction of the corresponding hypermatrix. The imaging data (e.g. T1 gadolinium enhanced MRI slices, PET slices etc.) including the definition of the tumour contour, its metabolically active sub‐regions and the anatomical structures of interest, the histopathological (e.g. type of tumour) and the genetic data (e.g. p53 status and other molecular data) of the patient are collected. The clinician delineates the tumour and the anatomical structures of interest by using a dedicated computer tool. In the case of radiotherapy, the planned distribution of the absorbed dose (e.g. in Gy) in the region of interest is also acquired. For the purpose of the 3D reconstruction and visualization of both the initial tumour and the simulation outcome, the 3D visualization package AVS/Express 4.2 has been used, (for details concerning the use of AVS/Express in the simulation model refer to Stamatakos et al).^18^ The description of the biological activity of the tumour is implemented by introducing the notion of the “geometrical cell (GC)”, the elementary cubic volume of the 3D discretizing mesh covering the region of interest as previously mentioned.

We assume that each GC of the mesh initially and normally accommodates a Number of Biological Cells (NBC). However, the maximum number of biological cells that is allowed to be accommodated within a GC is assumed to be NBC + [a fraction of NBC]. NBC apparently depends on the selected size of the GC and determines the quantization error of the model. The fraction of NBC considered in the code executions in this paper was 1/10 as this was shown to be the optimal one from both the convergence and CPU time demands points of view.^24^ Typical clonogenic cell densities are 10^4^ to 10^5^ cells/mm^3^.^4^ Since most GBM tumours are poorly differentiated and rapidly growing, we assume a clonogenic cell density of 2 × 10^5^ cells/mm^3^ in the proliferating cell region, 10^5^ cells/mm^3^ in the G_0_ cell region and 0.2 × 10^5^ cells/mm^3^ in the dead cell region of the tumour.

It is noted that each multidimensional element of the proposed hypermatrix has a clear physical and/or biological meaning (e.g. number of biological cells within a given phase, number of therapy hit cells residing in a given phase etc.). Therefore, the hypermatrix (such as the exemplary one outlined above) is used to describe the distribution of several critical physical and biological quantities over space and time.

### 6.2. Operator based presentation of the simulation model basics

In the following the various processes constituting the entire simulation algorithm are briefly and separately described with reference to the corresponding operators.

#### 6.2.1. Operator *f^T^*

Time is discretized and incremented. One hour has been adopted as the unit of time since 1h is the approximate duration of mitosis, the shortest cell cycle phase. According to the prescribed radiotherapy scheme the specific instants corresponding to the delivery of a radiation dose to the tumour region are defined. In each time step the geometrical mesh is scanned and the updated state of a given GC is determined on the basis of a number of behaviour algorithms:

#### 6.2.2. Operator *f^O^*

During each scan of the discretization mesh the effect of the oxygen and nutrient provision on the cells of each geometrical cell is taken into account. This provision, determining the metabolic potential of a region, is based on the imaging data and determines the distribution of the tumour cells in the proliferative, dormant and necrotic states within the regions without taking into account the eventual therapeutic interventions effects. Furthermore, distribution of the cells over the cell cycle phases (G_1_, S, G_2_, Mitosis) is considered based on experimental evidence (Katzung (Ed),^37^).

#### 6.2.3. Operator *f^H^*

(I) At the time instants corresponding to the delivery of a specific radiation dose to the tumour (according to the prescribed radiotherapy scheme and the acquired distribution of the absorbed dose in the region of interest) the number of cells killed in a particular GC is calculated based on the Linear Quadratic (LQ) Model, which is widely used in the pertinent literature.^5^,^7^,^38^,^39^ The fraction of cells surviving from a radiation dose D is given by

(21) $$\text{S}(\text{D})=\text{exp}[-(\alpha \text{D}+\beta {\text{D}}^{2})]$$

where α (Gy^−1^) and β (Gy^−2^) characterize the initial slope and the curvature, respectively, of the survival curve.

In an untreated tumour simulation case, the dose D would be set to zero.

(II) Lethally damaged cells following exposure to radiation undergo two mitotic divisions prior to death and disappearance from the tumour.^39^

Note: Any eventual molecular perturbators of the cell surviving fraction are to be incorporated into this operator.

#### 6.2.4. Operator *f^C^*

At each time step the time registers of all GCs are incremented by one hour. Cell loss due to apoptosis and necrosis is computed. According to the cytokinetic model appearing in^23^ possible transitions of the cells within a GC include: G_1_→S (if time spent in the G_1_ cell cycle phase ≥ TG_1_; TG_1_ = duration of the G_1_ phase), S→G_2_ (if time spent in the S cell cycle phase ≥ TS; TS=duration of the S phase), G_2_→M (if time spent in the G_2_ cell cycle phase ≥ = TG_2_; TG_2_ duration of the G_2_ phase), M→G_1_ or M→G_0_ (if time spent in the M cell cycle phase ≥ TM; TM =duration of mitosis), G_0_→G_1_ (if adequate oxygen and nutrient provision has been re‐established) or G_0_→N (if time spent in the G_0_ cell cycle phase ≥ TG_0_; TG_0_=maximum duration of the G0 phase before a cell enters necrosis). The previously mentioned durations of the cell cycle phases TX, X ∈ {G_1_, S, G_2_, M, G_0_} seem to follow the normal distribution according to pertinent literature. As a first approximation, we use the mean values of the duration of each cell cycle phase and neglect standard deviations. However pseudo‐random number generators are used in order to de‐synchronize the equivalence classes throughout the tumour.

The cell cycle duration T_C_ has been taken equal to 40 h. This is the average of the cell cycle durations we have found in the literature for GBM cell lines.^40^,^41^ In Katzung^37^ the approximate percentage of the cell cycle time spent in each phase by a typical malignant cell is assumed as follows: TG_1_ = 0.4T_C_, TS = 0.39T_C_, TG_2_ = 0.19T_C_, TM = 0.02T_C_. The duration of the G_0_ phase is taken to be TG_0_=25 h.^42^

The cell loss factor (CLF) is considered equal to 0.3.^43^ In 44 the authors note that cell loss is mainly due to necrosis (CLFN) and apoptosis (CLFA) and that gliomas have a low CLF in general. We assume that the total CLF (0.3) is the sum of the CLFN (0.27) and CLFA (0.03). We hypothesize low levels of apoptotic cells for GBM, as we have found that this is in general the case for gliomas.^35^,^36^,^44^

#### 6.2.5. Operator *f^E^*

**Figure 2:**
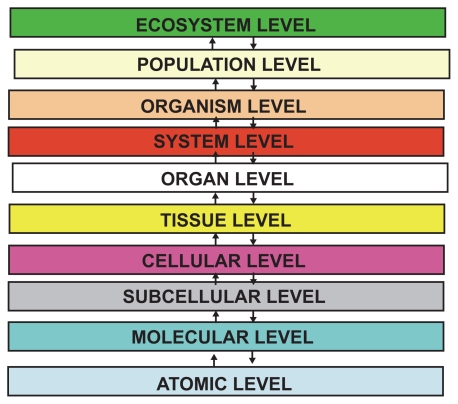
The proposed ten levels of biocomplexity.

**Figure 3:**
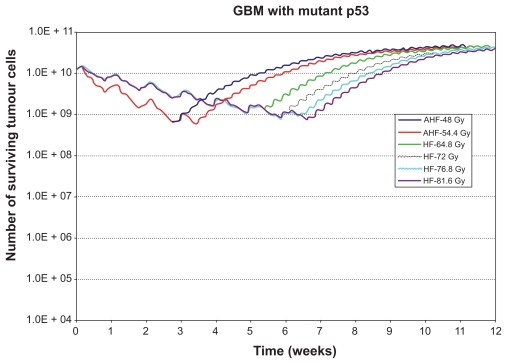
Number of surviving tumour cells as a function of time for the glioblastoma tumour with mutant p53 gene (see section 6). The radiotherapeutic schemes correspond to schemes considered by the rTOg 83-02 clinical study. **Abbreviations:** AhF, accelerated hyperfractionation; hF, hyperfractionation.

**Figure 4:**
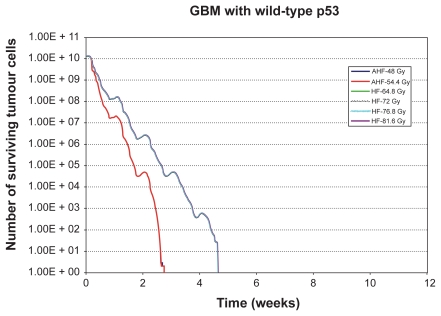
Number of surviving tumour cells as a function of time for the glioblastoma tumour with wild-type p53 gene (see section 6). The radiotherapeutic schemes correspond to schemes considered by the rTOg 83-02 clinical study. The curves on the right correspond to the hF schedules whereas the curves on the left correspond to the AhF schedules. **Abbreviations:** AhF, accelerated hyperfractionation; hF, hyperfractionation.

The differential tumour expansion and shrinkage algorithms are based on the use of random number generators in conjunction with adequately formed morphological rules. These rules lead to tumour shrinkage or expansion conformal to the initial shape of the tumour (if the mechanical properties of the surrounding normal tissue are considered uniform around the tumour and the tumour is not in contact with practically undeformable tissues such as bone). Two versions of the expansion and shrinkage algorithms have been tested. First version (a): For each GC, one out of the six possible directions of shrinkage or expansion is randomly chosen (Cartesian coordinate system XYZ centered at the current GC. Each axis defines two possible directions of movement). Second version (b1) Shrinkage: The outermost tumour GC is detected along each one of the six possible directions of shrinkage (Cartesian coordinate system XYZ centered at the current GC. Each axis defines two possible directions of movement). Its “6‐Neighbour” GCs belonging to the Tumour (NGCT) are counted. The direction corresponding to the maximum NGCT is finally selected out of the six possible directions as the direction along which the shifting of the GCs will take place (shifting direction). In case that more than one shifting directions have the same maximum NGCT, then the selection is based on the use of a random number generator. Second version (b2) Expansion: A similar, though inverse, morphological—mechanical rule can be applied in the case of tumour expansion. The need for the formulation of the second improved version of the tumour shrinkage and expansion algorithm has arisen from the inspection of the macroscopic results of the simulation algorithms. Specifically, the completely random selection of one out of the six possible shifting directions, according to the first version, results in a premature extensive fragmentation of the tumour region in case of radiotherapy, which is usually incompatible with clinical experience. The general trend is a conformal shrinking of most solid tumours (Perez and Brady,^45^ Figs. 1–4, p. 10). Using the second version of the algorithms this problem is solved. The mechanical properties of the surrounding normal tissue are considered uniform around the tumour, with the exception of an absolute lack of deformability of the bone. As a first approximation immunological reactions, invasion and formation of metastases have been ignored.

#### 6.2.6. Operator *f^U^*

After having completed a scan of the discretizing mesh the oxygen and nutrient field is updated based on the criterion determining the relative position of the proliferative, dormant and necrotic regions of the tumour. The reason for this process is to take into account any eventual expansion or shrinkage of the tumour that would lead to a perturbation of the previous metabolic potential field

## 7. Results

**Figure 5:**
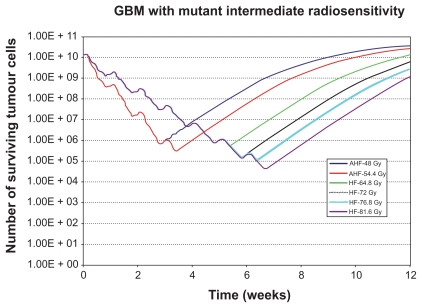
Number of surviving tumour cells as a function of time for the glioblastoma tumour with intermediately adjusted radiosensitivity parameters (see section 6). The radiotherapeutic schemes correspond to schemes considered by the rTOg 83-02 clinical study. **Abbreviations:** AhF, accelerated hyperfractionation; hF, hyperfractionation.

In order to clarify the actual effect of the composite discrete operator *f* introduced above the following indicative simulation predictions are included in this paper. Figures 3, 4 and 5 present the results of the *in silico* experiments in the form of the number of surviving tumour cells (proliferating and dormant) as a function of time, for the tumours with mutant p53, wild‐type p53 and the tumour with intermediate radiosensitivity, correspondingly. Improved tumour control following high‐dose HF irradiation is evident in the diagrams and is in agreement with the conclusions of the clinical study RTOG 83‐02. In fact, the higher the total dose in an HF schedule, the better the result in terms of tumour cell kill. It should be noted that the clinical study compared the treatments based on patient survival whereas the simulation results presented refer to tumour responsiveness. Although a correspondence of patient survival with tumour responsiveness may not always hold on a single patient basis, when taking into account relatively large clinical trial populations such a correspondence becomes much more reasonable.

More specifically, the inspection of the simulation results reveals that AHF schedules, which employ a higher fraction dose compared to HF schedules, seem at first to be beneficial as they achieve the maximum tumour cell kill at some instant. Nevertheless, the duration of the AHF schedules is smaller; as a result, if they fail in eradicating “all” tumour cells, tumour repopulation begins earlier (as in the cases of the tumour with mt p53 and the tumour with intermediate radiosensitivity). Only in the case of the tumour with wt p53, do all radio‐therapy schemes kill all the clonogenic cells we have initially assumed (although this may not be the case in reality due to considerable instabilities of the simulation when it comes to the last few living tumour cells (chaotic behaviour limits)), so the tumour does not regrow after the end of the treatment. Of course, regions of potential microscopic disease have not been considered, and the accuracy of the simulation model decreases as the number of tumour cells is reduced.

## 8. Discussion

The introduction of operator notation into the process of modeling malignant tumour response to therapeutic schemes has led to a brief and comprehensive description of the major steps of the simulation process. In this way highly complex algorithmic treatments are decomposed into simpler procedures which are readily identifiable by the wider research community. The use of mathematical symbols to denote complex algorithmic processes is expected to function as a stimulant for the advancement of multilevel biological modeling through *symbolic* mathematical expressions. Such expressions could by themselves provide hints for further questions, investigations and optimizations due to their inherent logical and quantitative associations. Especially the analogy of the operator notation in biomedicine with their use in several fields of physics such as classical and quantum mechanics can act as a source of guidance and quantitative insight into the hypercomplex biological phenomena.

Obviously the treatment presented should be viewed only as an initial step of a rather long term modeling process as more and more experimental and clinical knowledge could be incorporated into the models which are under continuous development and optimization. Specific aspects that are currently addressed in parallel include i.a. the mitotic potential categories of the cancer stem, progenitor and differentiated cells (leading to a new dimension in the discrete abstract space),^28^,^29^ adjacent normal tissue response,^46^ molecular networks (adapting the cell survival probability to the patient individualized context), chemotherapy optimization^20^,^21^,^28^,^29^ etc. A possible application of the approach presented in this paper is on the development of the *in silico* oncology action of the European Commission (EC) funded project “ACGT: Advancing Clinicogenomic Trials on Cancer” (FP6‐2005‐IST‐026996). In particular the development of the software simulation tool named “Oncosimulator”^47^ can be described based on the notation proposed in this paper. An analogous simulation tool is being developed within the framework of the EC funded project “ContraCancrum: Clinically Oriented Translational Cancer Multilevel Modelling” (FP7‐ICT‐2007‐2‐223979).

## 9. Conclusions

The introduction of the proposed hypermatrix and operator notation in order to denote, decompose and identify complex biological mechanisms that contribute to the hypercomplex phenomenon of malignant tumour growth and response to therapeutic schemes has been presented through the use of a discrete mathematics simulation model concerning radiotherapy response. Several aspects of the model had been developed by our research group in the past. Symbolic operator notation has provided a compact way of describing the most crucial simulation steps thus offering a possible basis for quantitative multilevel biology based on discrete mathematics. Furthermore, simulation results mimicking branches of the RTOG 83‐02 clinical study have provided both clarification of the actual content of the composite discrete operator and additional evidence for the potential of the simulation approach presented.
